# Persistent Effect of Advanced Glycated Albumin Driving Inflammation and Disturbances in Cholesterol Efflux in Macrophages

**DOI:** 10.3390/nu13103633

**Published:** 2021-10-17

**Authors:** Carlos André Minanni, Adriana Machado-Lima, Rodrigo Tallada Iborra, Lígia Shimabukuro Okuda, Raphael de Souza Pinto, Monique de Fátima Mello Santana, Aécio Lopes de Araújo Lira, Edna Regina Nakandakare, Maria Lúcia Cardillo Côrrea-Giannella, Marisa Passarelli

**Affiliations:** 1 Laboratório de Lípides (LIM-10), Hospital das Clínicas (HCFMUSP) da Faculdade de Medicina da Universidade de São Paulo, São Paulo 01246-000, Brazil; carlosminanni@gmail.com (C.A.M.); adrianasaldiba@gmail.com (A.M.-L.); rtiborra@yahoo.com.br (R.T.I.); ligia.okuda@gmail.com (L.S.O.); rspinto@usp.br (R.d.S.P.); moniquemello4@gmail.com (M.d.F.M.S.); aeciolira@hotmail.com (A.L.d.A.L.); enakonda@usp.br (E.R.N.); 2 Hospital Israelita Albert Einstein (HIAE), São Paulo 05652-900, Brazil; 3 Programa de Pós-Graduação em Ciências do Envelhecimento, Universidade São Judas Tadeu, São Paulo 03166-000, Brazil; 4 Programa de Pós-Graduação em Educação Física, Universidade São Judas Tadeu, São Paulo 03166-000, Brazil; 5 Laboratório de Carboidratos e Radioimunoensaio (LIM-18), Hospital das Clínicas (HCFMUSP) da Faculdade de Medicina da Universidade de São Paulo, São Paulo 01246-000, Brazil; maria.giannella@fm.usp.br; 6 Programa de Pós-Graduação em Medicina, Universidade Nove de Julho, São Paulo 01525-000, Brazil

**Keywords:** advanced glycated albumin, diabetes mellitus, cholesterol efflux, ABCA-1, inflammation, atherosclerosis

## Abstract

Advanced glycated albumin (AGE-albumin) impairs cholesterol efflux and contributes to inflammation in macrophages. The current study evaluated: (1) the persistence of the deleterious effect of AGE-albumin in cholesterol efflux and in inflammation, and (2) how metabolic control in diabetes mellitus (DM) contributes to attenuate the deleterious role of AGE-albumin in macrophage cholesterol homeostasis. Methods: AGE-albumin was produced in vitro or isolated from uncontrolled DM subjects’ serum before (bGC) and after improved glycemic control (aGC). Albumin samples were incubated with bone marrow-derived macrophages and ^14^C-cholesterol efflux or LPS- induced cytokine secretion were determined immediately, or after cell resting in culture media alone. The ABCA-1 degradation rate was determined after cell incubation with cycloheximide, and ABCA1 protein level by immunoblot. Oil Red O staining was used to assess intracellular lipid accumulation. Results: A persistent effect of AGE-albumin was observed in macrophages in terms of the secretion of inflammatory cytokines and reduced cholesterol efflux. HDL-mediated ^14^C-cholesterol efflux was at least two times higher in macrophages treated with aCG-albumin as compared to bGC-albumin, and intracellular lipid content was significantly reduced in aGC-albumin-treated cells. As compared to bGC-albumin, the ABCA-1 protein content in whole cell bulk was 94% higher in aCG-albumin. A 20% increased ABCA-1 decay rate was observed in macrophages treated with albumin from poorly controlled DM. AGE-albumin has a persistent deleterious effect on macrophage lipid homeostasis and inflammation. The reduction of AGEs in albumin ameliorates cholesterol efflux.

## 1. Introduction

Advanced glycation end products (AGEs) are prevalent in diabetes mellitus (DM) and independently predict cardiovascular disease [1]. During hyperglycemia, glycation occurs by the Maillard reaction that takes place via non-enzymatic interactions between reducing sugars and the amino-terminal portion of lysine and arginine residues in proteins, phospholipids, and nucleic acids. An unstable Schiff base is initially formed, leading to an Amadori Product, which undergoes irreversible conversion into AGEs. This process is intermediate by highly reactive oxoaldehydes formed by glucose autoxidation and rearrangements of the Schiff base and Amadori Product. Oxoaldehydes and dicarbonyl sugars, including methylglyoxal, glyoxal, 3-deoxyglucosane, and glycolaldehyde (GAD) are much more reactive than glucose and promote rapid modification of macromolecules by AGEs, including short and middle half-life proteins. Besides hyperglycemia, oxoaldehydes can be generated by the oxidation of polyunsaturated fatty acids and some amino acids, as well as in inflammatory reactions mediated by myeloperoxidase. Moreover, tobacco and dietary AGEs are exogenous sources of the Maillard reaction, which occurs according to food composition, cooking time, and temperature—adding an important source of AGEs to the human body [2].

AGEs are very heterogeneous compounds, although carboxymethyl lysine (CML) has been consistently found in association with atherosclerotic lesions. AGEs bind to the receptor for AGE (RAGE), eliciting nuclear factor KB (NFKB) activation and the expression of genes involved in vascular damage and inflammation [3]. Serum albumin is the major serum protein modified by glycation and positively correlates to tissue damage such as in the eyes, kidneys, and vessels [3]. Advanced glycated albumin (AGE-albumin) drawn from poorly controlled type 1 and type 2 DM subjects or modified in vitro by incubation with glycolaldehyde (GAD-albumin) is greatly modified by CML, pyrraline and argypirimidine, and disturbs reverse cholesterol transport (RCT). RCT is considered an antiatherogenic system mediated by HDL subclasses that drive excess cholesterol from peripheral cells—including arterial wall macrophages—to the liver, allowing its secretion into bile as free cholesterol or bile acids, and further excretion in feces. Moreover, by considering its ability to receive cholesterol from triglyceride-enriched lipoproteins during postprandial lipolysis, HDLc increases in plasma and helps to limit cholesterol supply to the arterial wall in a process recently described as reverse remnant-cholesterol transport (RRT) [4].

The ATP-binding cassette transporter A-1 (ABCA-1) plays a major role in the first step of the RCT, exporting cholesterol to lipid-poor apoA-I and pre-beta HDL [5]. Besides this, the ATP-binding cassette transporter G-1 (ABCG-1) exports cholesterol and oxysterols to larger HDL molecules, synergistically improving RCT.

AGE-albumin primes macrophages to inflammation elicited by S100-calgranulins and lipopolysaccharides (LPS) [6] and severely reduces ABCA-1 protein levels, reducing cholesterol efflux, and increasing intracellular accumulation of cholesterol and toxic oxysterols [7,8,9]. Considering previous evidence that AGEs favor inflammation and impair cholesterol efflux, the present study investigated how persistent the effect of AGE-albumin is in priming macrophages for inflammation and impairing cholesterol efflux. For this purpose, macrophages were challenged with AGE-albumin; after removal of the insult, they rested in a control medium for different time intervals, followed by the determination of cholesterol efflux or challenge with LPS to quantify the secretion of inflammatory cytokines. Besides this, in an attempt to test the mechanistic hypothesis that a better glycemic control in DM subjects contributes to attenuate the deleterious role of AGE-albumin in macrophage inflammation and cholesterol homeostasis, experiments were conducted with serum albumin drawn from uncontrolled DM subjects before and after improved metabolic control. We observed a long-lasting effect of AGE-albumin in inducing inflammation and cholesterol efflux impairment. On the other hand, the reduction of albumin modification by AGEs attained after improving DM control recovered ABCA-1 levels and cholesterol efflux, contributing to reduced intracellular lipid accumulation.

## 2. Experimental Section

### 2.1. In Vitro Modification of Bovine Albumin by Advanced Glycation

Bovine fatty acid free albumin (40 mg/mL) was modified in vitro by advanced glycation by incubation with freshly prepared 10 mM glycolaldehyde (Sigma Chem. Com. St. Louis, MO, USA) in phosphate buffer saline (PBS; (137 mmol/L NaCl; 4 mmol/L Na_2_HPO_4_; 2 mmol/L KCl; 1 mmol/L K_2_PO_4_, EDTA, pH = 7.4). Control albumin (C-albumin) was incubated with PBS only. Incubations were done for 4 days, at 37 °C, under sterile conditions and nitrogen atmosphere in a water bath shaker in the dark. After extensive dialysis against PBS containing EDTA, samples were sterilized through 22 µm filter. Samples contained < 50 pg endotoxin/mL as determined by the chromogenic Limulus amebocyte assay (Cape Cod, Falmouth, MA, USA).

### 2.2. Isolation of Plasma Lipoproteins

Low-density lipoprotein (LDL; d = 1.019–1.063 g/mL) and high-density lipoprotein subfraction 2 (HDL_2_; d = 1.063–1.125 g/mL) and subfraction 3 (HDL_3_; d = 1.125–1.21 g/mL) were isolated by discontinuous density ultracentrifugation of healthy human plasma donors. After dialysis, all samples were sterilized through a 22 µm filter and utilized within two weeks. Protein was measured by the Lowry technique [10]. Acetic anhydride was utilized to acetylate LDL as previously described [11], and these modified lipoproteins utilized to load macrophages with cholesterol. HDL subfractions were utilized as cholesterol acceptors in incubations with bone marrow-derived macrophages and J774 cells.

### 2.3. Culture of Bone Marrow-Derived Macrophages (BMDMs)

L929 cells (ATCC, American Tissue Culture Collection) were cultured in low-glucose DMEM (Gibco, Grand Island, NY, USA) plus 10% heat-inactivated fetal calf serum (Cultilab, Campinas, Brazil) and 1% penicillin/streptomycin (Gibco) for 7 days. The medium was stored at −20 °C (first week medium) until experiments, as a source of colony-stimulating factor-1 required for bone marrow cell differentiation into macrophages. Confluent monolayers were cultured with fresh medium for 7 more days in order to generate a second batch of conditioned medium (second week medium). Undifferentiated bone marrow cells were obtained from C57BL/6 wildtype mice. The femora and tibias were cleaned, and the end of each bone was cut off and filled with bone marrow medium (containing low glucose DMEM, penicillin/streptomycin, heat-inactivated fetal calf serum and L929-cell conditioned medium). Bone marrows were expelled with a jet of medium directed into a 50 mL screw top tube. Cell aggregates were broken up after gentle aspiration of the marrow; then cells were centrifuged for 6 min at 1000 rpm at room temperature. Cells were suspended in bone marrow medium and plated in culture dishes for 5 days at 37 °C under a 5% (v/v) CO_2_. The conditioned medium was changed for a new bone marrow medium and on the 6th day, the growth medium was completely changed to low glucose DMEM, 1% penicillin/streptomycin and 10% heat-inactivated fetal calf serum.

### 2.4. Culture of J774 Macrophages

J774 macrophages were grown in RPMI (Gibco) supplemented with 10% fetal bovine serum (Cultilab), 100 µg/mL streptomycin, 100 U/mL penicillin and 2 mM glutamine (Gibco) until confluence.

### 2.5. Measurement of Cholesterol Efflux from Macrophages

BMDMs were overloaded with acetylated LDL (50 µg/mL) and 3 µCi/mL of ^14^C-cholesterol (Amersham Biosciences, UK), for 24 h. After two washes with PBS containing fatty acid free albumin (FAFA), macrophages were kept for 48 h in DMEM (Gibco) containing 1% penicillin/streptomycin supplemented with 1 mg/mL of albumin isolated from DM and C subjects. HDL subfractions (50 µg/mL) were added after cell washing as cholesterol acceptors for 6 h incubation. Medium was collected and centrifuged to remove cell debris and cells were extracted with hexane/isopropanol (3:2; v:v). After solvent evaporation, the radioactivity was determined as well as ^14^C-cholesterol efflux—determined as ^14^C-cholesterol in the medium/^14^C-cholesterol in the medium + ^14^C-cholesterol in cells × 100. Values were subtracted from those obtained in incubations with DMEM/FAFA alone (basal efflux) in order to specifically determine the intrinsic ability of HDL subfractions in mediating cholesterol efflux [7].

### 2.6. Evaluation of the Persistence Time of the Effect of AGE-Albumin on Macrophage Inflammation

To determine whether the effect of AGE-albumin in sensitizing macrophages to inflammation persists after time, even after its removal from cell culture medium, macrophages overloaded with acetylated LDL (50 µg/mL) were incubated for 48 h with control-albumin, AGE-albumin or albumin isolated from subjects with DM bGC and aGC (1 mg/mL). Cells were carefully washed and maintained in DMEM/FAFA for a long time (0 h, 2 h, 4 h, 6 h, 8 h, 12 h and 24 h), and then incubated with LPS (1 μg/mL) for 24 h. The concentration of pro-inflammatory cytokines (IL-6, TNF) was determined by ELISA (R&D System—Duo Set, Minneapolis, MN, USA).

### 2.7. Evaluation of the Persistence Time of the Effect of AGE-Albumin on Macrophage Cholesterol Efflux

The persistence of albumin´s effect on macrophage cholesterol efflux mediated by HDL subfractions was determined in macrophages overloaded with acetylated LDL (50 µg/mL) and ^14^C-cholesterol (3 µCi/mL) that were incubated for 48 h with AGE-albumin or albumin isolated from subjects with DM bGC and aGC (1 mg/mL). After careful washing, cells were incubated with DMEM/FAFA alone for different periods of time (0 h, 3 h, 9 h and 12 h) and after incubated with HDL_2_ or HDL_3_ as cholesterol acceptors, for 6 h. The percentage of cholesterol efflux was determined as describe above.

### 2.8. Isolation of Serum Albumin from Subjects with DM before and after Glycemic Control

Subjects with type 1 (T1DM) and type 2 (T2DM) DM were recruited at the Hospital das Clínicas da Faculdade de Medicina da Universidade de São Paulo and non-DM control subjects at Faculdade de Medicina da Universidade de São Paulo. All participants were informed about the study and signed an informed written consent including approval for the publication of any potentially identifiable data included in this article. Subjects with microalbuminuria, uncontrolled thyroid disease, chronic kidney or liver disease, or other chronic disease, using antioxidants, current smokers or alcohol abusers were not included. Glycemia, triglycerides (TG), total cholesterol (TC), HDL cholesterol (HDLc), fructosamine, and albumin were determined in plasma by enzymatic techniques after overnight fasting. HbA_1c_ was determined by high-performance liquid chromatography (HPLC).

A physician intensively followed subjects with poorly controlled DM; diet and physical activity were prescribed as usual and maintained during the follow-up; drugs (insulin, metformin and gliclazide) were adjusted when necessary to assure better metabolic control. Other drugs such as statins, angiotensin-converting-enzyme inhibitor (ACEi), angiotensin 1 receptor blocker and L-thyroxine were used by some subjects and are described in Table 1. In the day before blood sample collection, subjects were advised to keep regular meals. Blood was drawn after overnight fasting before (bGC; *n* = 6) and after the improvement of glycemic control (aGC; *n* = 6), and serum was immediately isolated. Another group of poorly controlled T1DM subjects (*n* = 4) and non-DM control subjects (*n* = 5) was also included in the study, and blood samples were obtained only once as described above. Preservatives were added to serum (µL/mL of serum): 0.25% chloramphenicol/gentamicin (20 µL), of 5% sodium azide (10 µL; Merck, Darmastadt, Germany); 2 mM benzamidine (5 µL), 0.5 % aprotinin (5 µL) and phenyl methyl sulfonyl fluoride diluted in 30 mM dimethylsulfoxide (0.5 µL; Sigma-Aldrich, Steinheim, Germany) and samples were kept frozen at −80 °C until processing.

### 2.9. Isolation of Serum Albumin from Subjects with DM and Control Individuals

Albumin was isolated from all subjects’ serum by fast protein liquid chromatography (FPLC) using a HiTrap^TM^Blue (GE Healthcare, Uppsala, Sweden) affinity column, and purified by alcoholic extraction, as previously described [6]. Endotoxin levels in all samples were less than 50 pg of endotoxin/mL (Limulus Amebocyte Lysate; LAL, Cape Cod, Falmouth, MA, USA) and previous assays showed no cell toxicity induced by isolated albumins. Sample integrity was analyzed by mobility in acrylamide gel chromatography and was shown to be similar to commercially available standard human albumin (Sigma Chemical Co., St. Louis, MO, USA).

### 2.10. Determination of Carboxymethyllysine in Albumin Isolated from Human Serum and Modified In Vitro

Carboxymethyllysine (CML) was determined in albumin samples purified from human serum and in bovine albumin modified in vitro by advanced glycation by ELISA (Circulex CML, Woburn, MA, USA).

### 2.11. Measurement of Intracellular Lipid Content

BMDMs were incubated with DMEM/FAFA (*Low Glucose,* Gibco) containing acetylated LDL (50 μg/mL) for 48 h. After washing, cells were treated for 18 h with albumin isolated from subjects with DM before and after improvement of GC. Then, cells were incubated with these albumins in the presence or absence of HDL_2_ (50 μg/mL; 6 h). Cells were washed with PBS/FAFA and fixed with 10% formalin solution, for 1 h, at room temperature. Macrophages were stained with Oil Red O solution (Sigma-Aldrich, Brazil; 0.05% propylene glycol solution; Sigma-Aldrich) for 2 h, followed by washing with distilled water. The culture plates were then placed at 32 °C for 45 min. The coverslips were placed on slides in a jelly glycerin solution, and cell staining was quantified by a single-blinded investigator using an optical microscope (Sony CCD Camera/Olympus Microscope BX-51) and Image Pro Plus Media Cybernetics software (Bethesda, MD, USA). Ten fields of each slide were analyzed at 400 x magnitude. Data were expressed as the stained area detected by the software as a percentage of the total area.

### 2.12. Measurement of ABCA-1 Decay Rate

ABCA-1 degradation was analyzed in J774 macrophages that were incubated in 60 mm plates for 18 h in the presence of 10 µM LXR-agonist (T0901317; Sigma) to maximize ABCA-1 expression. After washing, cells were incubated with C or DM albumin (2 mg/mL) in the presence of 400 µg of cycloheximide (CHX; Sigma) for 0, 4, 8 and 12 h. CHX is an inhibitor of protein synthesis that allows the measurement of remaining ABCA-1 in whole cell extracts, reflecting its decay rate. After immunoblotting, ABCA-1 bands were corrected for beta-actin; data were normalized with time 0 as 100%. To calculate the ABCA-1 decay rate, we used the slope of the linear regression from time 0 to the end of incubation.

### 2.13. Determination of ABCA-1 Protein Level by Immunoblot

J774 cells were treated with albumin isolated from subjects with DM before and after improvement of GC (2 mg/mL) for 48 h. After washing, cells were scrapped with PBS and the cell pellet obtained after centrifugation (15,000 rpm, for 5 min at 4 °C). Samples diluted in TBS containing 1.5 mM aprotinin, pepstatin 2 μM, 1 μM leupeptin and 0.1 mM PMSF were sonicated for 12 s and centrifuged at 15,000 rpm for 15 min at 4 °C. Forty to 60 μg of sample were applied into a 6 or 7.5% T sodium dodecyl sulfate (SDS) polyacrylamide gel for electrophoresis. Unoccupied sites of PVDF membranes were blocked by incubation with PBS containing 5% skimmed milk and 0.05% Tween. Anti-ABCA-1 antibody (1:50; Novus Biologicals) and anti-rat antibody conjugated to peroxidase (1:2000; Life Technologies, Carlsbad, CA, USA) were utilized and bands obtained after reaction with ECL (WESTAR; Cyanagen, Bologna, Italy). Image capture was performed using the ImageQuant 350 (GE Healthcare, Piscataway, NJ, USA) and blots were quantified by densitometry (ImageQuant TL, Amersham Biosciences UK Limited). The densities of the respective lanes stained by beta actin were used for normalization and results were expressed as arbitrary units, related to the mean of the controls, which was set as 1.0.

### 2.14. Statistical Analysis

Statistical analysis was performed using GraphPad Prism 7 (GraphPad Software, Inc. 2017, San Diego, CA, USA). One-way ANOVA with Dunnett’s post-test or Student t test were utilized to compare results (mean ± SE). A *p*-value < 0.05 was considered statistically significant.

## 3. Results

**Figure 1:**
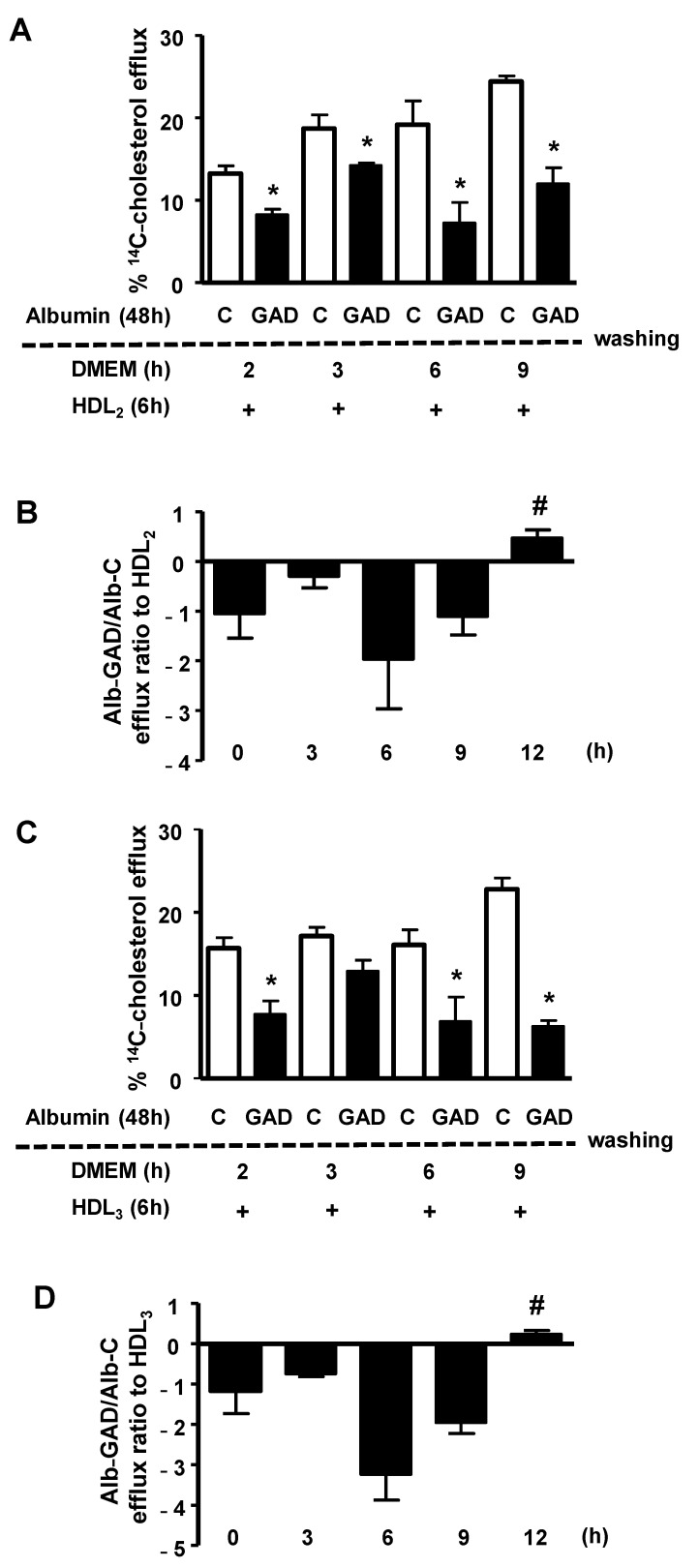
Persistence of the effect of GAD-albumin on ^14^C-cholesterol efflux impairment in macrophages. ^14^C-cholesterol and acetylated LDL overloaded BMDMs were incubated for 48 h with low-glucose DMEM containing 1 mg/mL medium of control (C-albumin) or glycolaldehyde-treated albumin (GAD-albumin). After this period, cells were washed with PBS/FAFA and immediately tested for cholesterol efflux mediated by HDL_2_ (panels **A** and **B**) or HDL_3_ (panels **C** and **D**), or after resting in DMEM/FAFA for different periods of time (0 to 12 h). Efflux experiments were performed for 6 h. In panels B and D, the efflux rate is expressed as ratio of GAD-albumin/C-albumin, considering three independent experiments. Comparisons were done by Student’s *t* test * *p* < 0.05 as compared to C-albumin (*n* = 3); # *p* < 0.05 as compared to other time periods.

The persistence of the effect of AGE-albumin in impairing macrophage cholesterol efflux was investigated by treating BMDMs, previously overloaded with acetylated LDL and ^14^C-cholesterol, with bovine serum albumin modified in vitro by AGEs (GAD-albumin), or control albumin. After the removal of albumins from the medium, cells were carefully washed and immediately tested for cholesterol efflux, or after resting for different periods in DMEM/FAFA. As shown in Figure 1, the percentage of ^14^C-cholesterol efflux mediated by both HDL_2_ (panel A) and HDL_3_ (panel C) was lower in cells treated with GAD-albumin in comparison to those treated with control albumin, and this effect of GAD-albumin persisted for up to 9 h. Considering data from three independent experiments (panels B and D) we observed that the cholesterol efflux rate (expressed as the ratio of GAD-albumin/C-albumin) was recovered only after 12 h after the removal of GAD-albumin from cells, confirming a long-lasting effect of AGE-albumin in impairing cholesterol export in macrophages.

**Figure 2:**
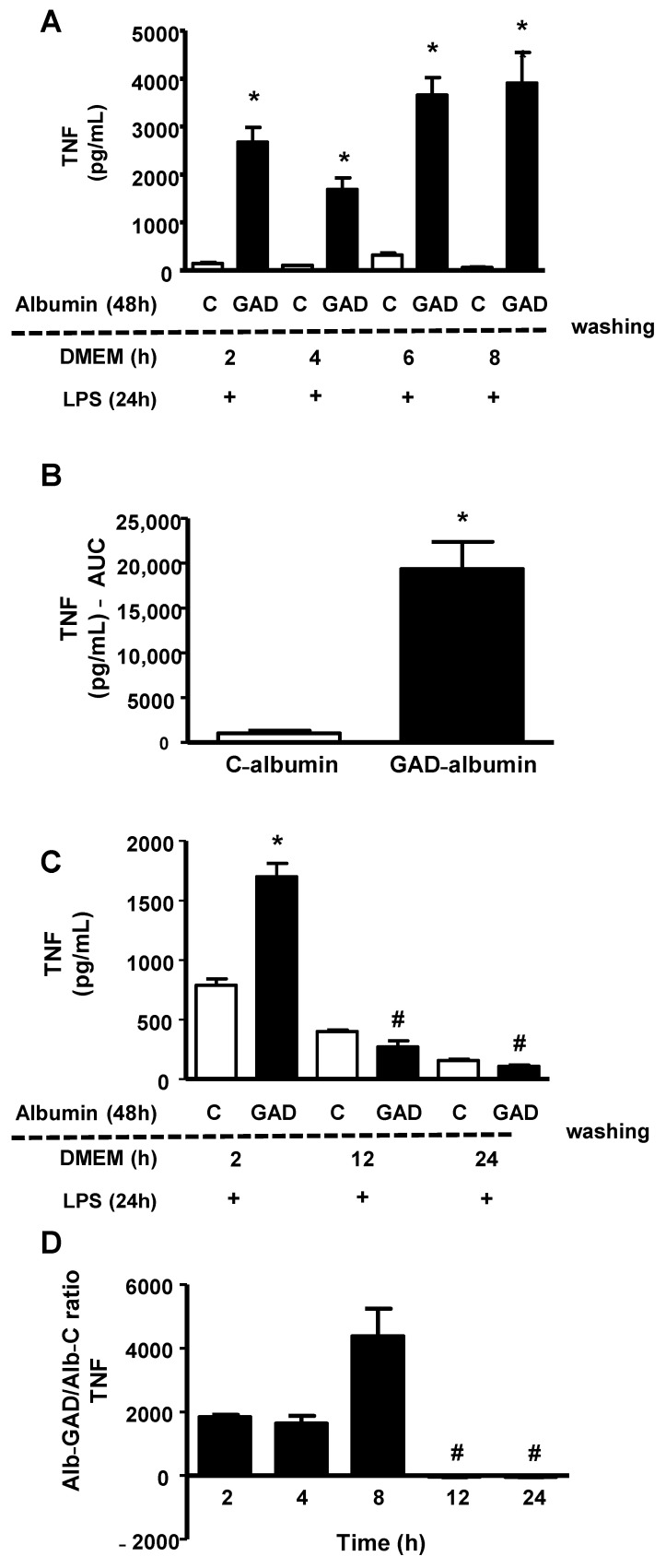

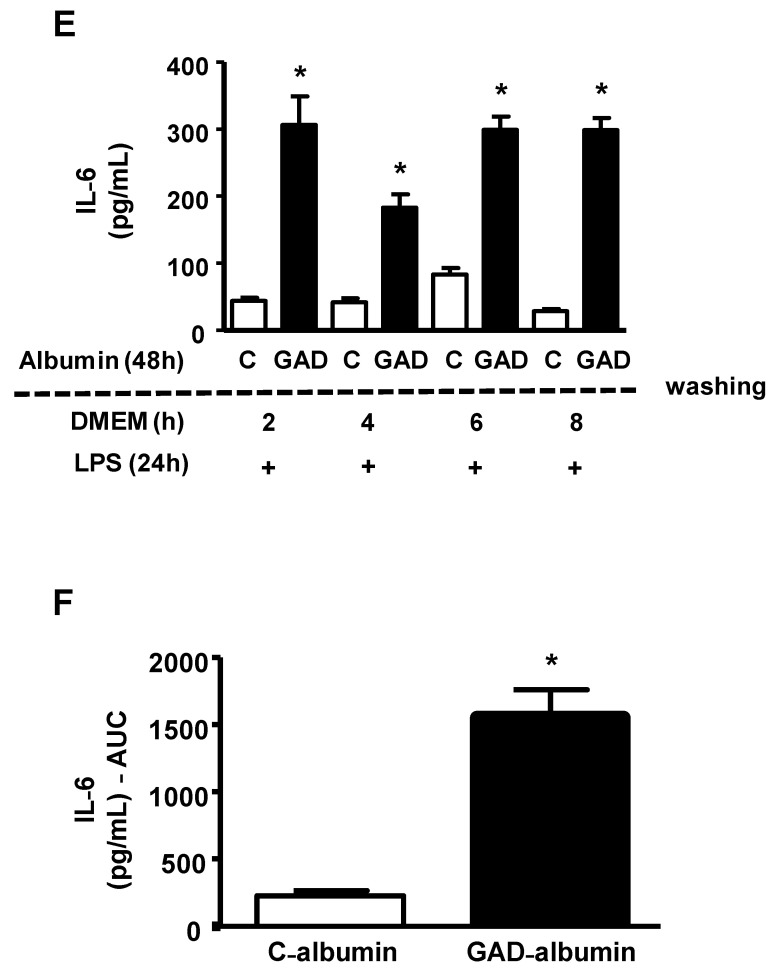
Persistence of the effect of glycolaldehyde (GAD)-modified albumin on the secretion of TNF and IL-6 by LPS-challenged macrophages. Cholesterol-overloaded BMDMs were incubated with low-glucose DMEM containing C or GAD-albumin (1 mg/mL), for 48 h. After this period, cells were washed with PBS/FAFA and incubated with DMEM/FAFA alone for different periods of time. After treatment with lipopolysaccharide (LPS, 1 μg/mL), for 24 h, TNF and IL-6 concentration were determined in the medium by ELISA. (Panel **A**): TNF secretion after 2 h, 4 h, 6 h, and 8 h of cell resting in DMEM/FAFA prior to LPS challenge (absolute values); (panel **B**): area under the curve (AUC) of TNF secretion; (panel **C**): TNF secretion (absolute values) after 2 h, 12 h, and 24 h of cell resting in DMEM/FAFA prior to LPS challenge; (panel **D**): independent experiments (from panels **A** and **C**) were considered together and expressed as the Alb-GAD/Alb-C ratio for TNF secretion; (Panel **E**): IL-6 secretion (absolute values); (panel **F**): area under the curve (AUC) of the IL-6 secretion along time. Comparisons were done by Student’s t test. * *p* < 0.05 in comparison to C-albumin (*n* = 3); # *p* < 0.05 in comparison to other time periods.

Since cholesterol accumulation favors inflammation and inflammatory stress disturbs cholesterol efflux, creating a vicious cycle, we analyzed how long the effect of GAD-albumin persists in LPS-challenged macrophages. Cholesterol-overloaded BMDMs were incubated for 48 h with GAD-albumin and C-albumin, rested for different intervals of time in DMEM/FAFA and then were challenged with LPS, for 24 h. As shown in Figure 2 (panel A), the secretion of TNF was higher in cells pretreated with GAD-albumin as compared to C-albumin and this was observed up to 8 h after the removal of GAD-albumin from the cell medium. These results provide support for GAD-albumin sensitization of macrophages to inflammation. A greater area under the curve (AUC) was obtained in GAD-albumin treated cells as compared to those treated with C-albumin (Figure 2, panel B). In another independent experiment dealing with different periods of time, we observed that the reduction in TNF secretion was observed only after 12 h of cell resting in the absence of GAD-albumin (Figure 2, panel C). Considering the experiments together, a reduction in the secretion of TNF expressed as the ratio of GAD-albumin/C-albumin was attained only after 12 h of GAD-albumin removal from cells (Figure 2, panel D). Similarly, the secretion of IL-6 elicited by LPS was much higher in cells incubated with GAD-albumin, even after 8 h of resting following the GAD-albumin insult (Figure 2, panel E), with a higher AUC for IL-6 secretion in cells treated with GAD-albumin in comparison to C-albumin (Figure 2, panel F).

In order to evaluate the role played by in vivo advanced glycation in DM, four subjects with T2DM and two with T1DM were selected from the intensive glycemic control protocol. Anthropometric and biochemical data of all subjects are depicted in Table 1. Creatinine, hemoglobin, and albumin levels were in the normal range in all subjects in the basal period, ensuring the precision of the measurements of hemoglobin and fructosamine. NPH and regular insulin, metformin and gliclazide were utilized for glycemic control achievement, although as expected, the interval of time for DM control was quite different among subjects. The amount of carboxymethyllysine (CML) in albumin was decreased in all aGC individuals as compared to the previous period of inadequate glycemic control (bGC). DM metabolic control based on HbA1c and fructosamine levels was achieved in all subjects with DM, as showed in Table 1.

**Table 1:** Age, anthropometric and biochemical data of DM subjects on intensive glycemic control. Individual albumin samples isolated from serum before (bGC) and after improvement of glycemic control (aGC) were utilized for experiments as indicated.

Subject		1	2	3	4	5	6
DM type		1	1	2	2	2	2
Age (years)		51	33	46	43	54	27
Gender		M	M	M	M	F	F
BMI (kg/m^2^)		20.7	17.6	27	23.8	26.5	32.5
TC (mg/dL)		206	187	256	155	172	156
HDLc (mg/dL)		97	75	42	23	72	43
TG (mg/dL)		74	76	123	187	51	92
Glucose (mg/dL)	bGC	98	184	147	128	239	135
	aGC	103	174	138	60	86	98
HbA1c (%)	bGC	9.2	13.7	12.9	16.5	10.5	8.5
	aGC	7.0	7.5	6.1	6.7	8.1	7.1
Fructosamine (µmol/L)	bGC	390	493	325	408	444	287
	aGC	375	319	242	241	360	248
CML (mU/μg albumin)	bGC	50	14	40	98	24.9	13.3
	aCG	8	11.1	19	43.6	17.5	9.8
Insulin		+	+	+	+	+	+
Metformin		-	-	+	+	+	+
Gliclazide		-	-	-	+	-	+
ACEI or ARB		-	-	+	-	+	+
Statin		+	-	+	-	+	+
Time for DM compensation (months)		12	21	4	3	5	6

BMI: body mass index; TC: total cholesterol; TG: triglycerides; HDLc: HDL cholesterol; HbA1c: glycated hemoglobin; ACEI: angiotensin conversion enzyme inhibitors; ARB: angiotensin 1 receptor blockers.

**Figure 3:**
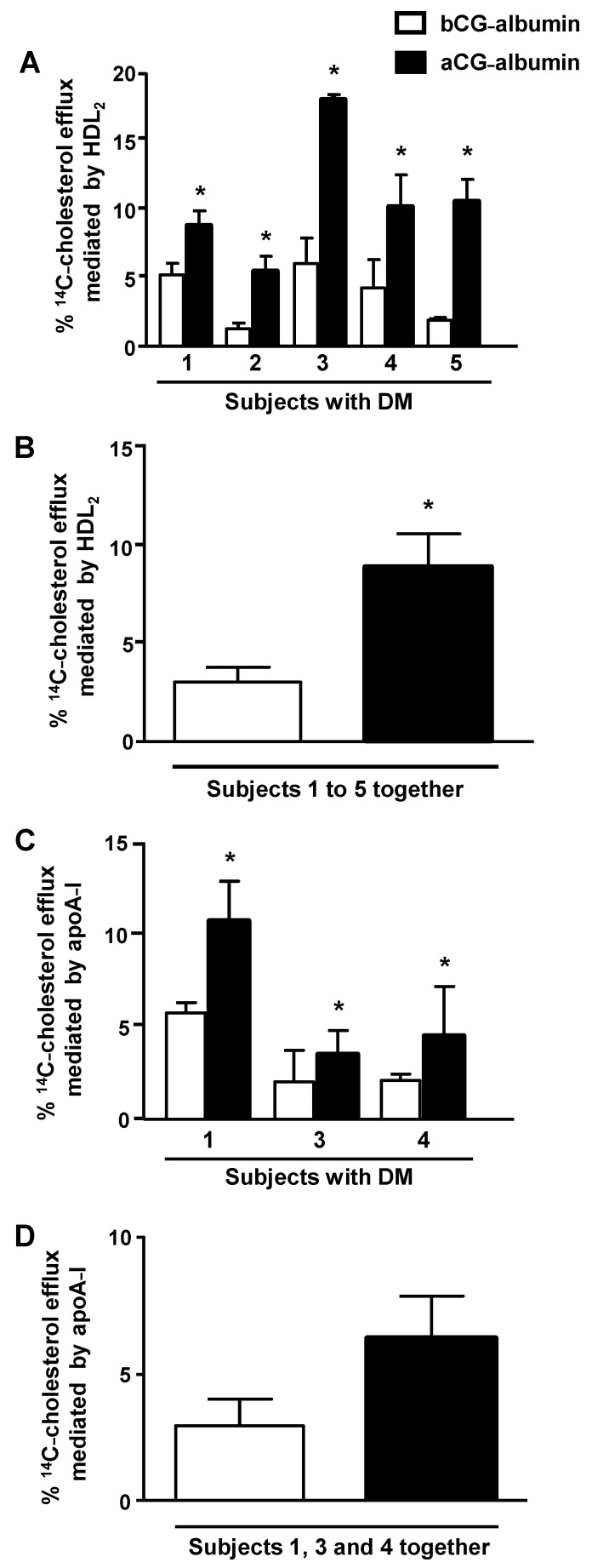
^14^C-cholesterol efflux from macrophages treated with serum albumin isolated from subjects with DM before (bCG) and after improvement of glycemic control (aGC). BMDMs were overloaded with acetylated LDL and ^14^C-cholesterol. After washing and equilibration in DMEM/FAFA, BMDMs were treated with 1 mg/mL of serum albumin isolated from subjects with DM bCG and aGC, for 48 h. After rinsing, cells were incubated with HDL_2_, for 6 h (panel **A**: subjects 1 to 5) or apoA-I for 8 h (panel **C**: subjects 1, 3 and 4). The % of cholesterol efflux was calculated as: ^14^C-cholesterol in medium/^14^C-cholesterol in medium + ^14^C-cholesterol in cell × 100. The specific efflux mediated by HDL or apoA-I was calculated by subtracting values of total efflux (incubations with DMEM/FAFA plus HDL_2_ or apoA-I) from those obtained in incubations with DMEM/FAFA alone (basal efflux). In panels **B** and **D**, data are presented with all subjects together, respectively, for efflux mediated by HDL_2_ and apoA-I. Comparisons were done by Student’s t test (mean ± SE; *n* = 4); * *p* < 0.05.

Due to sample limitation, albumins from subjects 1 to 5 were utilized to measure macrophage cholesterol efflux, and those from subjects 1, 3 and 6 in experiments to access ABCA-1 protein level. The cholesterol efflux mediated by HDL_2_ was increased in macrophages treated with albumin isolated from subjects with DM (subjects 1 to 5) with aGC as compared to the period of bad control, bGC (Figure 3, panel A). In Figure 3, panel B, data from subjects 1 to 5 are shown together, demonstrating that the improvement of GC was able to recover the ability of macrophages to export excess cholesterol to HDL_2_. The apoA-I-mediated cholesterol efflux from macrophages was tested with albumins isolated from subjects 1, 3 and 4. A higher cholesterol exportation was obtained when cells were treated with aGC-albumin in comparison to bGC-albumin (Figure 3, panel C), although no statistical significance was observed when subjects 1, 3 and 4 were depicted together (Figure 3, panel D).

**Figure 4:**
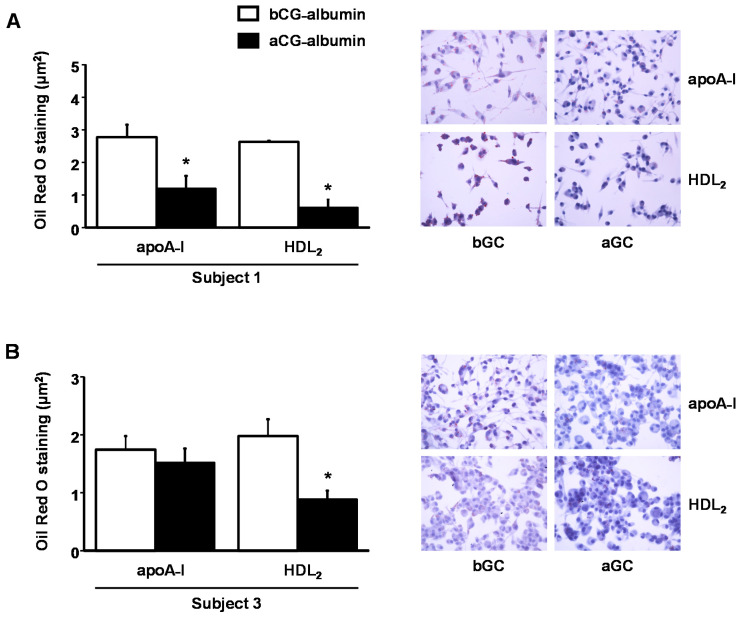
Intracellular lipid content in macrophages treated with serum albumin isolated from DM subjects before (bCG) and after improvement of glycemic control (aGC). BMDMs were overloaded with acetylated LDL and after washing treated with 1 mg/mL of serum albumin isolated from DM subjects bCG and aGC, for 18 h. Cells were rinsed and then incubated with apoA-I or HDL_2_ (6 h) as cholesterol acceptors. Intracellular lipid content was determined after staining with Oil Red O (representative photographs for each experimental condition). (Panel **A**): subject 1; (Panel **B**): subject 3. Comparisons were done by Student’s t test (mean ± SE; *n* = 4); * *p* < 0.05.

A significant reduction in intracellular lipid content was evidenced by the Oil Red O staining in cells incubated aGC-albumin and exposed to HDL_2_ cholesterol acceptors in comparison to cells incubated with albumin-isolated bGC, although the effect in apoA-I incubations was only observed in subject 1 [Figure 4, panel A (subject 1) and panel B (subject 3)].

**Figure 5:**
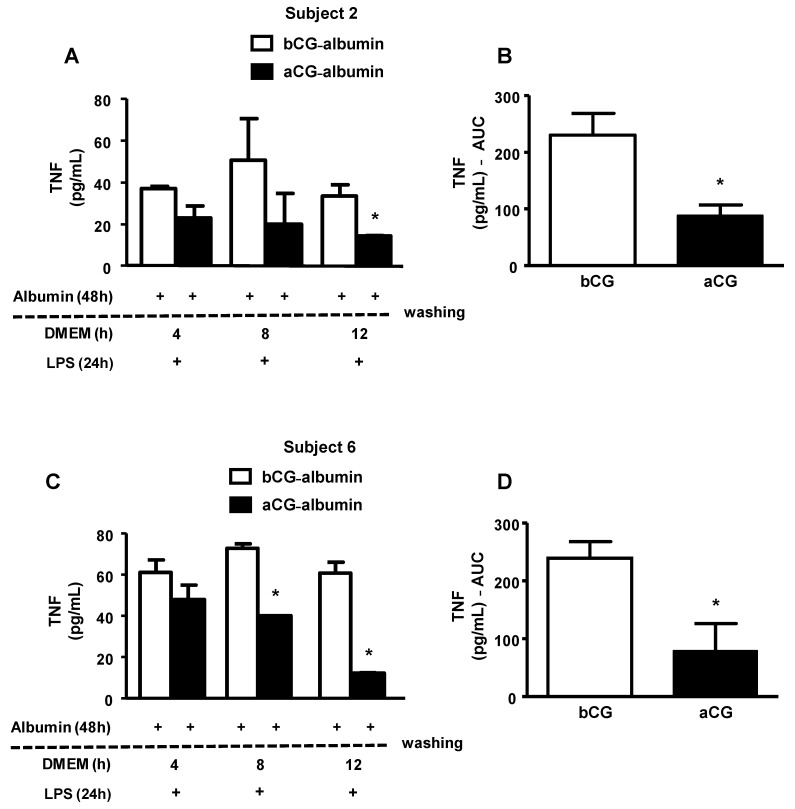
Persistence of the effect of albumin isolated before (bGC) and after (aGC) improvement of glycemic in subjects with DM in the inflammatory response elicited by LPS in macrophages. Cholesterol-overloaded BMDMs were incubated with low-glucose DMEM containing albumin isolated from subjects with DM (subjects 2 and 6) bGC and aGC (1 mg/mL), for 48 h. After this period, cells were washed with PBS/FAFA and incubated with DMEM/FAFA alone for different periods of time. After treatment with lipopolysaccharide (LPS 1 μg/mL), for 24 h, TNF concentration was determined in the medium by ELISA. Data are expressed as percentage in relation to incubations with control albumin. (Panels **A** and **C**): TNF secretion (absolute values) in incubations with albumins (bCG and aGC) from subjects 2 and 6, respectively. (Panels **B** and **D**): area under the curve (AUC) for TNF secretion in incubations with albumins (bGC and aGC) from subjects 2 and 6, respectively. Comparisons were done by Student’s *t* test * *p* < 0.05 in comparison to bCG (*n* = 3).

Cholesterol-overloaded BMDMs were incubated with albumin isolated from subjects with bGC and aGC DM (individuals 2 and 6). As shown in Figure 5, the persistence of the AGE-albumin’s effect on the secretion of TNF by macrophages was observed up to 8 h after removing DM albumins from the cell medium, and this effect was lower with aCG-albumin than with bCG-albumin. These results were in agreement with those obtained with GAD-albumin (Figure 2); it is then possible to assume that both in vitro and in vivo advanced glycated albumin exert long-lasting effects in macrophages, impairing lipid efflux and inducing inflammation.

**Figure 6:**
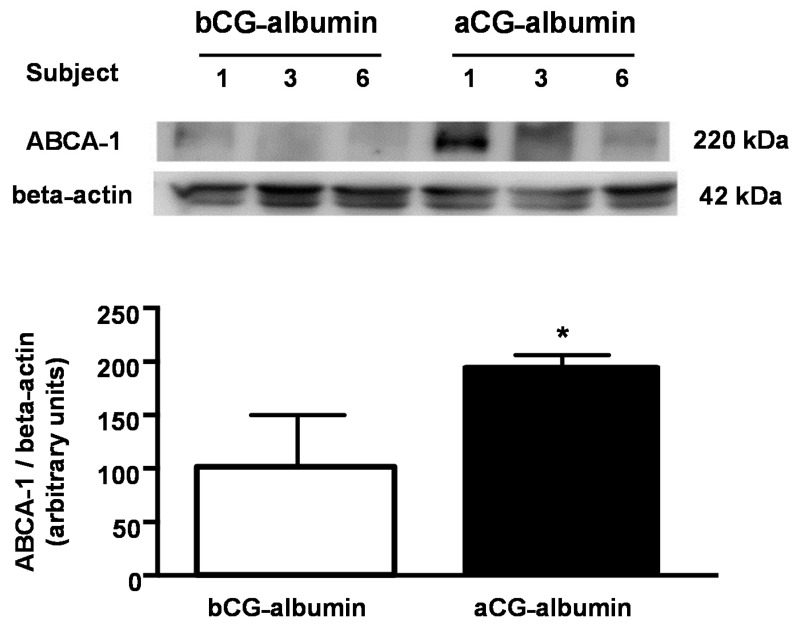
ABCA-1 protein level in macrophages treated with albumin isolated before (bGC) and after (aGC) improvement of glycemic control. ABCA1 protein levels and representative immunoblot. After 18 h-incubation with 2 mg/mL albumin isolated from subjects 1, 3, and 6 bGC and aGC, J774 macrophages were scrapped and equal amounts of cell protein loaded into a 6% polyacrylamide gel. Immunoblot was performed using anti-ABCA-1 antibody. Data are presented as arbitrary units (AU) corrected by beta-actin. Samples derived from the same experiment were processed in same gel/blot. Comparisons were done by Student’s t test (*n* = 3, mean ± SE). * *p* < 0.05.

In order to detail the role of DM-albumins in the modulation of ABCA-1, albumins isolated prior to and after the improvement in metabolic control were utilized to treat J774-macrophages and measure the ABCA-1 protein level by immunoblot. These cell lines were utilized as they had the required amount of cell protein to measure ABCA-1 content. As compared to bGC-albumin, the ABCA-1 protein content in whole cell bulk was 94% higher in macrophages treated with aGC-albumin (Figure 6).

In another set of experiments, a pool of albumin isolated from four poorly controlled T1DM subjects and from five non-DM control subjects (C) was utilized. Age, BMI and plasma lipids were similar between controls and T1DM. Fasting glucose, HbA1c and fructosamine levels were higher in T1DM. In agreement with this, the amount of CML was four times higher in DM-albumins as compared to C-albumins (Table 2).

**Figure 7:**
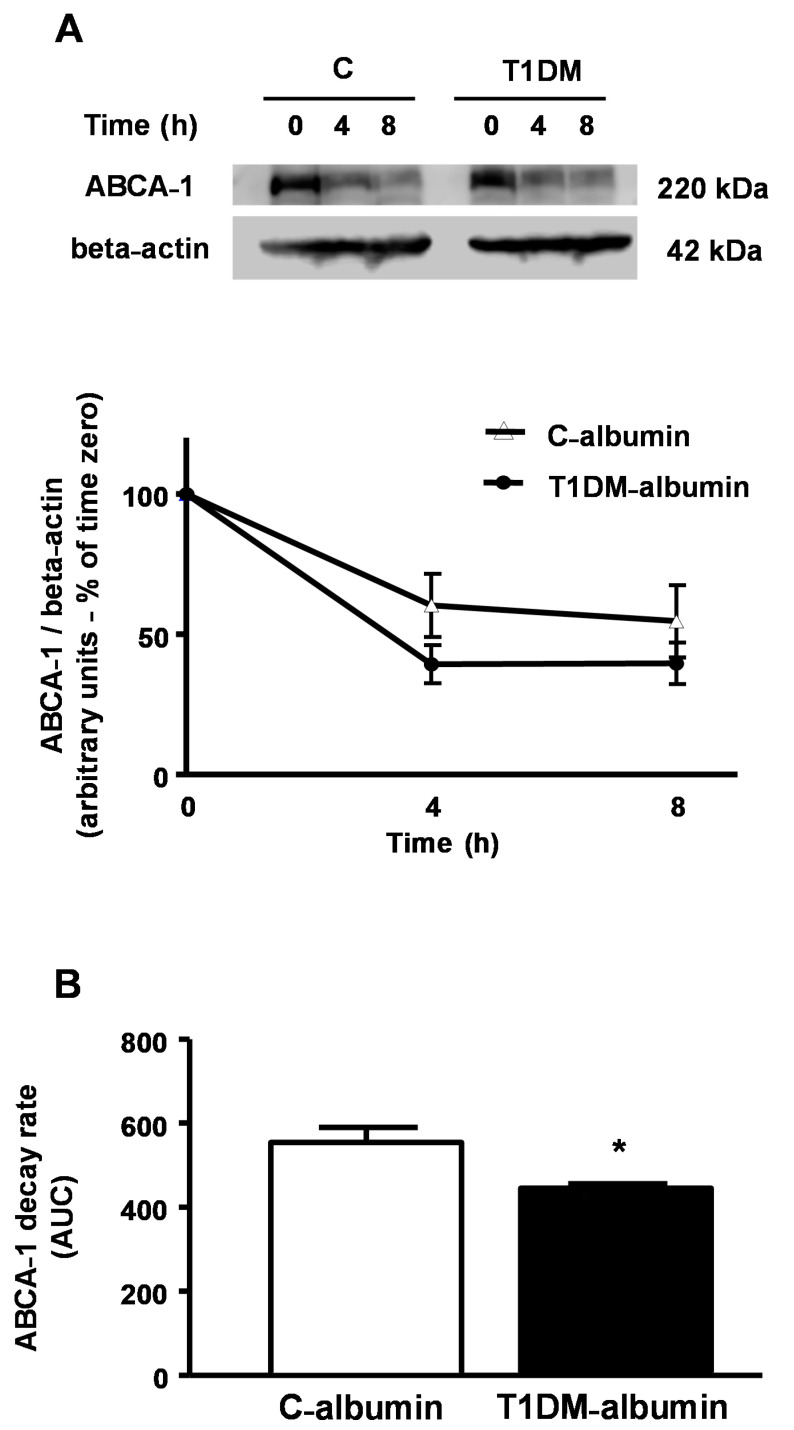
ABCA-1 decay rate in J774 macrophages treated with albumin isolated from poorly controlled T1DM and control subjects. J774 macrophages were incubated with T0901317 overnight and then exposed to a pool of serum albumin (2 mg/mL) isolated from non-DM control subjects (C; *n* = 5) or poorly controlled T1DM individuals (*n* = 4) in the presence of cycloheximide over time. Immunoblot was performed using anti ABCA-1 antibody and bands were corrected by beta-actin. Three independent experiments were performed, and a representative blot is shown in panel **A**. The ABCA-1 decay rate was calculated by the area under the curve (AUC) of ABCA-1 level (panel **B**). Comparisons were done by Student’s t test (*n* = 3, mean ± SE). * *p* < 0.05.

J774-macrophages were incubated over time with C and T1DM albumins (2 mg/mL) in the presence of cycloheximide (400 µg) to determine the ABCA-1 decay rate. A 20% increase in ABCA-1 decay rate was observed in macrophages treated with T1DM-albumin (Figure 7, panel A), with a lower AUC as compared to C-albumin (Figure 7, panel B). This result agrees with previous data showing that AGE-albumin reduces ABCA-1 protein levels by increasing ABCA-1 intracellular degradation, which can be reverted by the improvement of glycemic control.

## 4. Discussion

Long-term randomized clinical trials in both T1DM and T2DM subjects clearly showed that the precocity of intensive glycemic control prevents or delays the development of long-term complications [12,13]. The pathological basis for cellular and tissue damage relies on oxidative stress and AGEs, which modulate cell responses involved in metabolic memory. In addition, epigenetic changes alter the expression of genes contributing to metabolic memory-based injury [1,14]. Exogenous AGEs from dietary sources as well as endogenous AGEs are implicated in metabolic alterations related to atherogenesis [15,16]. In particular, it has previously been shown that AGEs impair RCT, largely due to damage by ABCA-1 and ABCG-1-mediated cholesterol efflux from macrophages.

The present study investigated the persistence of AGE-albumin in sensitizing macrophages to inflammation and in damaging cholesterol homeostasis. We demonstrated that: (1) advanced glycated albumin produced in vitro by incubation with glycolaldehyde (GAD-albumin) had long-lasting effects in macrophages that impaired cholesterol efflux and primed those cells to the secretion of inflammatory cytokines induced by LPS; (2) albumin modified in vivo by AGEs—isolated from DM subjects—also persistently damaged macrophage cholesterol efflux and induced inflammation, which was ameliorated by improving glycemic control, reflected by reductions in albumin modification by CML; (3) the reduction of albumin modification by AGEs restored ABCA-1 levels in macrophages. These results point to a role of AGE-albumin in metabolic cell memory, inducing inflammation and disturbing excess cholesterol removal from macrophages. Albumin is the main glycation-modified serum protein in DM, which is attributed to its high concentration in the circulation and 59 lysine residues in its structure, which makes its modification favorable during hyperglycemia and postprandially [17]. Albumin and lipoproteins are capable of transporting AGEs absorbed from AGE-containing diets, allowing their transport to tissues [18]. More recently, glycated albumin has been shown as being positively correlated to HbA1c and associated with coronary heart disease, ischemic stroke, heart failure, and death [19,20]. Besides this, glycated albumin reflects the risk of subclinical atherosclerosis assessed by the carotid intima-media thickness in middle-aged and elderly Chinese populations with impaired glucose regulation [21].

It has been demonstrated that both early and advanced glycation negatively affect lipid and lipoprotein metabolism. Particularly, AGEs impair cholesterol efflux by both LXR-dependent and independent mechanisms. Regarding ABCA-1, it is intriguing that AGE-albumin did not alter its levels of mRNA although severely it compromises ABCA-1 protein levels in macrophages. Cholesterol accumulation triggers inflammation, and in fact, AGE-albumin primes macrophages to the inflammatory response elicited by LPS and calgranulins. Here, we investigated how long the effects of AGE-albumin persist in macrophages. Cholesterol efflux was measured in cholesterol-loaded BMDMs, previously treated with GAD-albumin or serum albumin drawn from subjects with T1DM and T2DM bGC and aGC. Six subjects were independently analyzed according to reductions in HbA1c, fructosamine, and advanced glycation of albumin (CML content). GAD-albumin, as well as in vivo glycated albumin, impaired cholesterol efflux for a sustained period of time. In most DM individuals analyzed, macrophages treated with serum albumin isolated aGC presented higher values of cholesterol efflux to HDL and reduced intracellular lipid content in comparison to cells treated with albumin isolated during the period of bad glycemic control.

Serum albumin is modified by AGEs due to its reactions with oxoaldehydes, which are elevated in DM, mostly postprandially, and due to chronic inflammation. Elevated levels of oxoaldehydes are related to CV disease, myocardial infarction, and mortality in T2DM subjects [22]. Although the half-lives of hemoglobin and albumin are quite different, as well as the kinetics of early and advanced glycation reactions that characterize, respectively, HbA1c and CML-albumin formation, in all six individuals with DM a reduction in HbA1c was observed—accompanied by decreased levels of albumin derivatization by CML.

Interestingly, ABCA-1 protein levels were lower in cells treated with albumin isolated bGC as compared to aGC agreeing with the increased rate of ABCA-1 decay rate in macrophages of a different cell line exposed along time with albumin from subjects with poorly controlled T1DM as compared to albumin from control subjects.

In human umbilical vascular endothelial cells, human glycated albumin differentially regulates proteins involved in endothelial dysfunction, namely related to apoptosis and oxidative stress and the NF-KB cascade downstream to AGE-RAGE signaling, which can be counteracted by soluble RAGE [23]. The deleterious effects of AGE-albumin in cholesterol homeostasis are mediated by RAGE—a highly expressed multi-ligand receptor in atherosclerotic plaques—since they were not observed in macrophages from RAGE knockout mice [24]. In another study, BMDMs from mice with DM presented a reduced cholesterol efflux to apoA-I and HDL, as compared to BMDMs from control animals. Ager (RAGE) deletion improved cholesterol efflux to both acceptors [25].

In humans, Fadini et al. (2014) demonstrated that the reduction in HbA1c levels was correlated with HDLc increases, but HDL chemical modification by glycation as well as the improvement in the HDL function along the RCT was not investigated [26]. In this sense, the results showed in this study point to a direct and prolonged role of albumin glycation in the impairment of HDL function in the first step of the RCT, independently of HDL composition, size, and plasma levels.

AGE-albumin primes macrophages to inflammation, eliciting a greater secretion of inflammatory cytokines induced by lipopolysaccharide (LPS)—a classical inflammatory stimulus. Cholesterol accumulation in macrophages triggers inflammation, contributing to atherosclerotic lesion evolution. Besides this, the inflammatory stress downregulates ABCA-1-driven cholesterol exportation by creating a vicious cycle and triggering endoplasmic reticulum stress, inflammasome activation, and inducing cell apoptosis and pyroptosis that contribute to plaque rupture. Utilizing an experimental approach to investigate the persistence of the effects of AGE-albumin in macrophages, a persistent deleterious effect of albumin-GAD was observed up to 8 h after the end of exposure to AGE-albumin, inducing greater production of inflammatory mediators (TNF and IL-6) when compared to control-albumin, and causing a reduction in HDL_2_ and HDL_3_-mediated cholesterol efflux, which normalized 12 h after the end of exposure to GAD-albumin. Previous experiments had observed a 54% and 55% reduction in cholesterol efflux mediated by HDL_2_ and HDL_3_, respectively; however, this is the first demonstration of the persistence of this effect and recovery after resting in an AGE-free environment [7].

The nature of those long-lasting actions of AGE-albumin in macrophages was not addressed in this investigation, However, previous in vitro studies have shown the persistence of expression and activity of oxidative and inflammatory stress markers and mediators of cell death in endothelial and retinal cells treated for 14 days with high-glucose medium, followed by exposure for 7 days in low-glucose medium [14]. In addition, the generation of ROS induced by hyperglycemia favors persistent epigenetic changes in human endothelial cells and in animal models [3], characteristically due alterations in the proximal promoter region of the *NFKB* gene (p65 subunit), which promotes the expression of inflammatory genes. These changes (in general, in the histone methylation profile) persisted for six days after normalization of glucose concentration in the culture medium, and for months in animals with DM after recovery of pancreatic beta-cell function. Future investigations should deal with epigenetics in macrophages treated with glycated albumin.

Although other components of the metabolic syndrome that underlies T2DM can act as determinants of CVD, this is the first demonstration that serum albumin can modulate lipid flux in macrophages, and that this is under the direct influence of glycemic control in subjects with DM.

Limitations of the present study are the small number of DM individuals studied and the fact that we did not account for other modifications in albumin molecules apart from their advanced glycation, including drugs and other components carried by albumin, as well as the impact of albumin early glycation. Nonetheless, it was possible to confirm the specificity of advanced glycation in inducing cholesterol efflux damage, as previously related by others [7,24,25,27] as a proof of concept. According to the reference study [7] and considering the effect of the paired sample proportion on the statistical power of the study, from a mechanistic point of view, the results obtained in the present investigation show that the improvement of glycemic control in subjects with DM may contribute to preventing the negative impact of AGE-albumin in macrophage cholesterol efflux by reducing ABCA-1 decay rate and increasing ABCA-1 protein levels. Nonetheless, the long-lasting effect of glycated albumin in macrophages compromising cholesterol homeostasis in a cellular model of metabolic memory should be kept in mind. Due to sample limitations, we could not perform a statistical correlation between hemoglobin, fructosamine, and AGE levels with cholesterol efflux rate and other mechanistic variables that were measured in this study. Additionally, we could not establish a cut-off point in these parameters to obtain a better response of reverse cholesterol transport, which requires a large population study.

## 5. Conclusions

As a proof of concept, this investigation based on a cell culture model reinforces findings from clinical trials showing that the glycemic control, by reducing the modification of albumin by advanced glycation, may contribute to ameliorating cholesterol removal and inflammation in macrophages—helping to prevent the development of atherosclerosis in DM. Despite this observation, the long-lasting effects of AGE-albumin in macrophage intracellular signaling need to be detailed further. Considering the high amount of AGEs in DM, but also in processed foods and high-fat diets, and their generation independently of hyperglycemia, these data indicate an important role of AGEs in the damage of the reverse cholesterol transport that could lead to atherosclerosis.

## Figures and Tables

**Table 2:** Age, anthropometric and biochemical data of non-DM controls and subjects with poorly controlled T1DM.

	Controls (*n* = 5)	T1DM (*n* = 4)
Age (years)	28	30
BMI (kg/m^2^)	25	24.5
TC (mg/dL)	131	125
HDLc (mg/dL)	58	63
TG (mg/dL)	110	63
Glucose (mg/dL)	78	173 *
HbA1c	5.3	9.7 *
Fructosamine (μmol/L)	202	414 *
CML (mU/μg albumin)	5.8 ± 2.3	23 ± 11.9 *
Insulin	-	+

BMI: body index mass; TC: total cholesterol; TG: triglycerides; HDLc: HDL cholesterol; HbA1c: glycated hemoglobin; CML: carboxymethyllysine. * *p* < 0.05 in comparison to controls.

## Data Availability

All data reported are included in the manuscript and upon personal request to the authors raw data can be shared.

## References

[1] Brings S., Fleming T., Freichel M., Muckenthaler M.U., Herzig S., Nawroth P.P. (2017). Dicarbonyls and Advanced Glycation End-Products in the Development of Diabetic Complications and Targets for Intervention. Int. J. Mol. Sci..

[2] Uribarri J., Woodruff S., Goodman S., Weijing C., Chen X., Pyzik R., Yong A., Striker G.E., Vlassara H. (2010). Advanced Glycation End Products in Foods and a Practical Guide to Their Reduction in the Diet. J. Am. Diet. Assoc..

[3] Paneni F., Beckman J.A., Creager M.A., Cosentino F. (2013). Diabetes and vascular disease: Pathophysiology, clinical consequences, and medical therapy: Part I. Eur. Heart J..

[4] Kontush A. (2020). HDL and Reverse Remnant-Cholesterol Transport (RRT): Relevance to Cardiovascular Disease. Trends Mol. Med..

[5] Phillips M.C. (2014). Molecular mechanisms of cellular cholesterol efflux. J. Biol. Chem..

[6] Okuda L.S., Castilho G., Rocco D.D., Nakandakare E.R., Catanozi S., Passarelli M. (2012). Advanced glycated albumin impairs HDL anti-inflammatory activity and primes macrophages for inflammatory response that reduces reverse cholesterol transport. Biochim. Biophys. Acta.

[7] Machado-Lima A., Iborra T.R., Pinto R.S., Sartori C.H., Oliveira E.R., Nakandakare E.R., Stefano J.T., Giannella-Neto D., Corrêa-Giannella M.L.C., Passarelli M. (2013). Advanced glycated albumin isolated from poorly controlled type 1 diabetes mellitus patients alters macrophage gene expression impairing ABCA-1-mediated reverse cholesterol transport. Diabetes-Metab. Res. Rev..

[8] Machado-Lima A., Iborra T.R., Pinto R.S., Castilho G., Sartori C.H., Oliveira E.R., Okuda L.S., Nakandakare E.R., Gianella-neto D., Machado U.F. (2015). In Type 2 Diabetes Mellitus Glycated Albumin Alters Macrophage Gene Expression Impairing ABCA1-Mediated Cholesterol Efflux. J. Cell. Physiol..

[9] Iborra R.T., Machado-Lima A., Okuda L.S., Pinto P.R., Nakandakare E.R., Machado U.F., Correa-Giannella M.L., Pickford R., Woods T., Brimble M.A. (2018). AGE-albumin enhances ABCA1 degradation by ubiquitin-proteasome and lysosomal pathways in macrophages. J. Diabetes Complicat..

[10] Lowry O.H., Rosenbrough N.J., Farr A.L., Randall R.J. (1951). Protein Measurement with the Folin-Phenol Reagent. J. Biol. Chem..

[11] Basu S.K., Goldstein J.L., Anderson R.W., Brown M.S. (1976). Degradation of cationized low-density lipoprotein and regulation of cholesterol metabolismo in homozygous familial hypercholesterolemia fibroblastos. Proc. Natl. Acad. Sci. USA.

[12] UKPDS UK Prospective Diabetes Study Group (1998). Intensive blood-glucose control with sulphonylureas or insulin compared with conventional treatment and risk of complications in patients with type 2 diabetes (UKPDS 33). Lancet.

[13] Nathan D.M., Cleary P.A., Backlund J.Y., Genuth S.M., Lachin J.M., Orchard T.J., Raskin P., Zinman B. (2005). Diabetes Control and Complications Trial/Epidemiology of Diabetes Interventions and Complications (DCCT/EDIC) Study Research Group. Intensive diabetes treatment and cardiovascular disease in patients with type 1 diabetes. N. Engl. J. Med..

[14] Ihnat M.A., Thorpe J.E., Kamat C., Szabó C., Green D.E., Warnk L.A., Lacza Z., Cselenyák A., Ross K., Shakir S. (2007). Reactive oxygen species mediate a cellular ‘memory’ of high glucose stress signalling. Diabetologia.

[15] Prasad A., Bekker P., Tsimikas S. (2012). Advanced Glycation End Products and Diabetic Cardiovascular Disease. Cardiol. Rev..

[16] Gill V., Kumar V., Singh K., Kumar A., Kim J.J. (2019). Advanced Glycation End Products (AGEs) May Be a Striking Link Between Modern Diet and Health. Biomolecules.

[17] Stirban A., Tschöpe D. (2015). Vascular Effects of Dietary Advanced Glycation End Products. Int. J. Endocrinol..

[18] Koschinsky T., He C., Mitsuhashi T., Bucala R., Liu C., Buenting C., Heitmann K., Vlassara H. (1997). Orally absorbed reactive glycation products (glycotoxins): An environmental risk factor in diabetic nephropathy. Proc. Natl. Acad. Sci. USA.

[19] Kumeda Y., Inaba M., Shoji S., Ishimura E., Inariba H., Yabe S., Okamura M., Nishizawa Y. (2008). Significant correlation of glycated albumin, but not glycated haemoglobin, with arterial stiffening in haemodialysis patients with type 2 diabetes. Clin. Endocrinol..

[20] Selvin E., Rawlings A.M., Lutsey P.L., Maruthur N., Pankow J.S., Steffes M., Coresh J. (2015). Fructosamine and Glycated Albumin and the Risk of Cardiovascular Outcomes and Death. Circulation.

[21] Ma X., Shen Y., Hu X., Hao Y., Luo Y., Tang J., Zhou J., Bao Y., Jia W. (2015). Associations of glycated haemoglobin A1c and glycated albumin with subclinical atherosclerosis in middle-aged and elderly Chinese population with impaired glucose regulation. Clin. Exp. Pharmacol. Physiol..

[22] Hanssen N.M.J., Westerink J., Scheijen J.L.J.M., van der Graaf Y., Stehouwer C.D.A., Schalkwijk C.G., SMART Study Group (2018). Higher Plasma Methylglyoxal Levels Are Associated With Incident Cardiovascular Disease and Mortality in Individuals With Type 2 Diabetes. Diabetes Care.

[23] Yang G., Huang Y., Wu X., Lin X., Xu J., Chen X., Bai X., Li Q. (2018). Endogenous Secretory Receptor for Advanced Glycation End Products Protects Endothelial Cells from AGEs Induced Apoptosis. BioMed Res. Int..

[24] Machado-Lima A., López-Díez R., Iborra R.T., de Souza Pinto R., Daffu G., Shen X., Nakandakare E.R., Machado U.F., Corrêa-Giannella M.L.C., Schmidt A.M. (2020). RAGE Mediates Cholesterol Efflux Impairment in Macrophages Caused by Human Advanced Glycated Albumin. Int. J. Mol. Sci..

[25] Xu L., Wang Y.R., Li P.C., Feng B. (2016). Advanced glycation end products increase lipids accumulation in macrophages through upregulation of receptor of advanced glycation end products: Increasing uptake, esterification and decreasing efflux of cholesterol. Lipids Health Dis..

[26] Fadini G.P., Iori E., Marescotti M.C., de Kreutzenberg S., Avogaro A. (2014). Insulin-induced glucose control improves HDL cholesterol levels but not reverse cholesterol transport in type 2 diabetic patients. Atherosclerosis.

[27] Daffu G., Shen X., Senatus L., Thiagarajan D., Abedini A., Hurtado Del Pozo C., Rosario R., Song F., Friedman R.A., Ramasamy R. (2015). RAGE Suppresses ABCG1-Mediated Macrophage Cholesterol Efflux in Diabetes. Diabetes.

